# Targeting Myeloid-Derived Cells: New Frontiers in the Treatment of Non-alcoholic and Alcoholic Liver Disease

**DOI:** 10.3389/fimmu.2019.00563

**Published:** 2019-03-27

**Authors:** Luisa Vonghia, Mikhaïl A. Van Herck, Jonas Weyler, Sven Francque

**Affiliations:** ^1^Department of Gastroenterology and Hepatology, Antwerp University Hospital, Antwerp, Belgium; ^2^Laboratory of Experimental Medicine and Paediatrics, University of Antwerp, Antwerp, Belgium

**Keywords:** myeloid-derived cells, NAFLD (non-alcoholic fatty liver disease), ALD (alcoholic liver disease), treatment, liver immunology

## Abstract

Non-alcoholic fatty liver disease (NAFLD) and Alcoholic Liver Disease (ALD) are major causes of liver-related morbidity and mortality and constitute important causes of liver transplantation. The spectrum of the liver disease is wide and includes isolated steatosis, steatohepatitis, and cirrhosis. The treatment of NAFLD and ALD remains, however, an unmet need, and therefore it is a public health priority to develop effective treatments for these diseases. Alcoholic and non-alcoholic liver disease share common complex pathogenetic pathways that involve different organs and systems beyond the liver, including the gut, the adipose tissue, and the immune system, which cross-talk to generate damage. Myeloid-derived cells have been widely studied in the setting of NAFLD and ALD and are implicated at different levels in the onset and progression of this disease. Among these cells, monocytes and macrophages have been found to be involved in the induction of inflammation and in the progression to fibrosis, both in animal models and clinical studies and they have become interesting potential targets for the treatment of both NAFLD and ALD. The different mechanisms by which these cells can be targeted include modulation of Kupffer cell activation, monocyte recruitment in the liver and macrophage polarization and differentiation. Evidence from preclinical studies and clinical trials (some of them already in phase II and III) have shown encouraging results in ameliorating steatohepatitis, fibrosis, and the metabolic profile, individuating promising candidates for the pharmacological treatment of these diseases. The currently available results of myeloid-derived cells targeted treatments in NAFLD and ALD are covered in this review.

## Introduction

**Figure 1:**
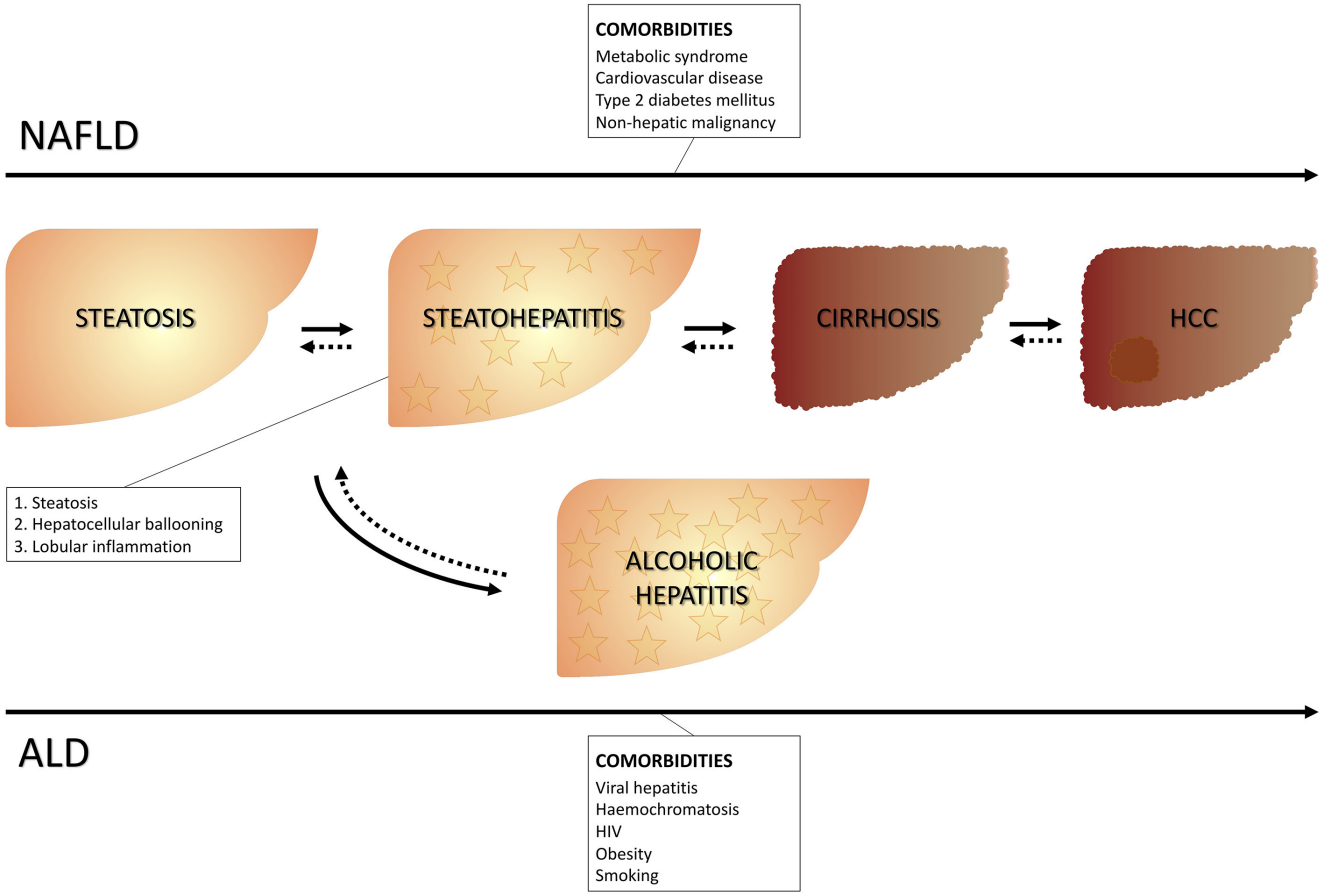
Clinical course of NAFLD and ALD. NAFLD, Non-alcoholic liver disease; ALD, Alcoholic liver disease.

Fatty liver represents a wide spectrum of disease encompassing stages ranging from isolated steatosis to steatohepatitis and it can be accompanied by different grades of fibrosis up to cirrhosis with all its complications, including hepatocellular carcinoma. The onset of fatty liver can occur in the presence or in absence of excessive alcohol consumption. The cut-off of a daily alcohol consumption ≥30 g for men and ≥20 g for women (1) is used to differentiate alcoholic vs. non-alcoholic fatty liver disease. Therefore, the presence of fatty liver identifies non-alcoholic fatty liver disease (NALFD) in the absence of excessive alcohol consumption, and alcoholic liver disease (ALD), in the presence of excessive alcohol consumption. The presence at liver histology of steatosis, as well as both lobular inflammation and hepatocyte ballooning specifically identifies steatohepatitis [respectively, non-alcoholic steatohepatitis (NASH) and alcoholic steatohepatitis (ASH), depending on whether or not there is an association with excessive alcohol consumption] (2) (Figure 1).

Although they share similar histologic characteristics, these two entities have different peculiarities. NAFLD can be associated to metabolic impairment and to cardiovascular disease and is considered the hepatic expression of the metabolic syndrome (3, 4) (Table 1).

**Table 1:** Definition of metabolic syndrome (3, 4).

**Required**	**Waist circumference ≥94/80 cm for men/women**
Number of abnormalities	≥2 of
Hypertension	≥130/85 mmHg or treatment for hypertension
Fasting glucose	≥100 mg/dl or treatment for type 2 diabetes
Triglycerides	>150 mg/dl or treatment for dyslipidaemia
HDL cholesterol	<40/50 mg/dl for men/women or treatment for dyslipidaemia

ALD, instead, is specifically related to alcohol-induced damage, including alcoholic hepatitis (AH), which represents a severe type of ASH, usually associated with more severe clinical course and histological lesions (5).

This dichotomy is, however, not always so unequivocal and appears—at least in part—arbitrary, given that patients consuming moderate amounts of alcohol may also have metabolic risk factors that predispose them to NAFLD and these metabolic factors seem to have a higher impact on the occurrence of steatosis and fibrosis (6).

The prevalence of NAFLD in the Western adult population is 25–30%, and even higher in populations with risk factors such as obesity or diabetes (7). About 20% of heavy drinkers develop fatty liver (8) and 35–40% of patients with chronic excessive alcohol abuse develop alcoholic hepatitis (AH) (9). ALD and NAFLD, respectively, represent the second and the third cause of liver transplantation and NAFLD has been estimated to become the primary cause of liver transplantation in the next decades (10).

Given the burden of these diseases, understanding the complex underlying mechanisms and the crosstalk between the different organs involved in the pathogenesis of NAFLD and ALD (and specifically of steatohepatitis) has been a research priority in the last decade, also in order to identify possible therapeutic targets.

The pathogenesis of NAFLD is complex and implicates the crosstalk between different metabolically active sites. Initially, the so called “two hits” hypothesis was proposed: insulin resistance, the “first hit,” leads to hepatic triglyceride accumulation (steatosis) and is followed by a “second hit” driven by, amongst others, oxidative stress, which in turn favors the development of steatohepatitis and fibrosis (11). Subsequent research has transformed this model into a “multiple parallel hits” hypothesis in which a number of different processes involving various organs such as adipose tissue, gut and muscle contribute to a cascade of inflammation, fibrosis and eventually tumorigenesis. In this setting, endoplasmic reticulum stress, cytokines, adipokines, and immunity are emerging drivers of liver damage (12).

The pathogenesis of ALD largely relates to the direct toxic effects of alcohol and its intermediate metabolite acetaldehyde. Together, these agents induce oxidative stress, mitochondrial damage, lipogenesis, hepatic fat accumulation—through increased influx of free fatty acids originating from the adipose tissue and gut-derived chylomicrons—, malnutrition, and leakage of endotoxins from the gut. Subsequently, these processes will result in the activation of a myriad of immune cells [including Kupffer cells (KC)] and the secretion of proinflammatory cytokines (13). [For an extensive review about the pathogenesis of, respectively, NAFLD and ALD see (13–16)].

**Figure 2:**
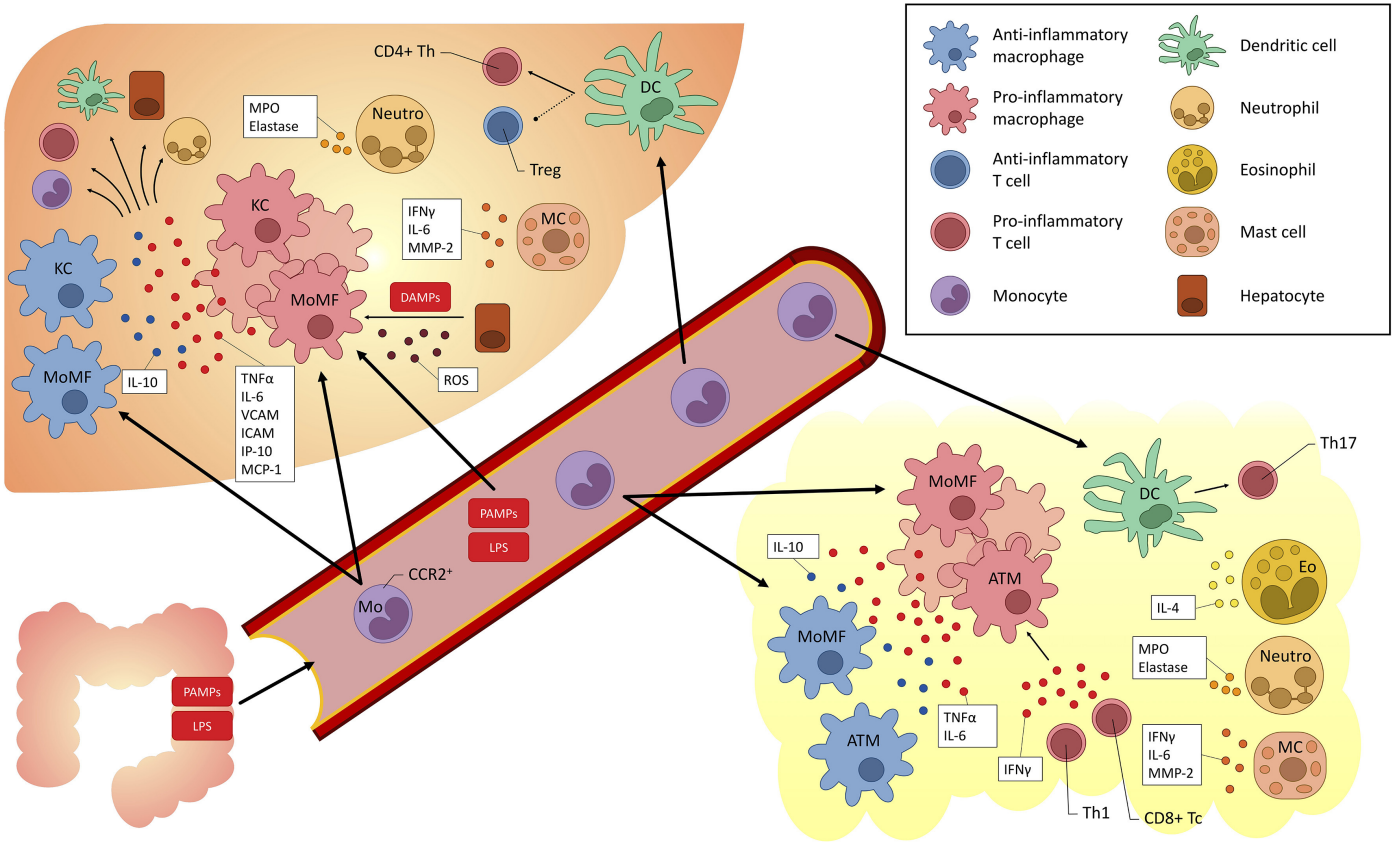
Overview of myeloid-derived cells implicated in fatty liver (alcoholic and non-alcoholic) and their cross-talk with other cells. KC, Kupffer cells; DC, dendritic cells; Eo, eosinophils; Neutro, neutrophils; MC, mast cells; Th, T helper; LPS, lipopolysaccharide; TLR, Toll-like receptor; TNF, tumor necrosis factor; IL, interleukin; IFN, interferon; ROS, reactive oxygen species; Tregs, T regulatory cells; MoMF, monocyte-derived macrophages; ATM, adipose tissue macrophages; MPO, myeloperoxidase; MMP, matrix metalloproteinase; VCAM, vascular cell adhesion molecule; ICAM, intercellular adhesion molecule; IP, interferon gamma-induced protein; MCP, Monocyte chemoattractant protein; PAMP, pathogen-associated molecular pattern; DAMP, danger-associated molecular pattern; CCR, C-C chemokine receptor; ROS, reactive oxygen species.

Moreover, the liver itself displays immune properties, and can be viewed as an “immunological organ” (14, 17, 18). Many immune cell populations have been studied and have been implicated in the pathogenesis of fatty liver (both alcohol and non-alcohol related) and may act as treatment targets (Figure 2).

Currently, no drugs are approved for the treatment of fatty liver, constituting an unmet medical need and a public health priority. Concerning possible treatment targets for fatty liver, several considerations should be noted. Firstly, a candidate target to block one or more pathways involved in the pathogenesis of the disease should be identified. Secondly, the “aim” of the treatment, i.e., reduction of either disease activity (i.e., steatohepatitis) or fibrosis progression, should be determined. Currently, the most desired outcome is still under debate. In the setting of NAFLD, while fibrosis has been identified as the most important predictor of both liver- and non-liver-related adverse outcomes [including overall and liver-related mortality (with a decline in prognosis from F2 onwards)], steatohepatitis is considered the driving force of these outcomes (19). This dichotomy, however, seems to be rather artificial, given that different pathways overlap and fibrosis progression is probably to be considered a marker of long-standing disease activity (and therefore a driver of the outcome). Considering ALD—aside from the fact that the cornerstone of any therapeutic intervention is alcohol abstinence—the same general concepts described above are true (20). Moreover, AH, steatosis, fibrosis itself, and especially alcoholic steatohepatitis are all independent predictive factors of fibrosis progression (1, 21).

## Myeloid-Derived Cells

Among the myeloid-derived cells (Table 2), monocytes and macrophages play an important role in the onset of both fatty liver and fibrosis. The liver harbors about 80% of all macrophages of the body and is also patrolled by other myeloid cells (such as blood monocytes), which scan the liver vasculature and eventually infiltrate into the liver. Monocyte-derived cells can develop into liver dendritic cells or monocyte-derived macrophages, the former being mainly responsible for antigen presentation of small or soluble structures to adaptive immune cells and the latter acting as primary filter cells, constantly removing particles from the circulation. KC are resident macrophages that belong to the reticuloendothelial system in the liver, which constitutes a primary line of defense against invading microorganisms, functions as a sensor for altered tissue integrity and largely contributes to maintain tissue homeostasis by contributing to the anti-inflammatory micromilieu as well as directly inducing tolerance in passenger leukocytes patrolling the sinusoidal system (22).

**Table 2:** Summary of the impairment of myeloid-derived cells in NAFLD and ALD.

	**NAFLD**	**ALD**
Monocytes	- Differentiation into tissue resident macrophages - Differentiation in DC	- Differentiation into tissue resident macrophages - Differentiation in DC
Macrophages/KC	- M1 enhancement - Imbalance of lipogenesis - Increased LPS/TLR4-mediated signaling - Increased TNF-α, IL-1β, IFN-γ, IL-6 - Fibrosis stimulation	- M1 enhancement - Increased LPS/TLR4-mediated signaling - Increased TNF-α, IL-1β, ROS
DC	- Altered CD8/CD4 ratio - Decreased Treg infiltration - Increased inflammation	- Increased cytokine secretion via TLRs - Increased TNF-α, IFN-γ
Neutrophils	- Liver infiltration - Progression to steatohepatitis (MPO)	- Liver infiltration - Increased TNF-α
Eosinophils	- Increased Th2-type cytokines - Increased M2 polarization	- Increased Th2-type cytokines - Increased M2 polarization

*NAFLD, Non-alcoholic liver disease; ALD, alcoholic liver disease; DC, dendritic cells; IFN, interferon; IL, interleukin; KC, Kupffer cells; LPS, lipopolysaccharide; NAFLD, non-alcoholic fatty liver disease; ROS, reactive oxygen species; Th2, T helper 2; TLR, Toll-like receptor; TNF, tumor necrosis factor; Treg, T regulatory cell; MPO, myeloperoxidase*.

Traditionally, macrophages were categorized dichotomically in either pro-inflammatory (M1) or anti-inflammatory (M2) phenotypes. These cells, however, display a broad spectrum of activation states in which macrophages often perform multiple functions and simultaneously express “M1” and “M2” markers (23).

Macrophages critically influence not only liver inflammation but also metabolic impairment (namely insulin resistance) in metabolic disorders and alcoholic liver disease (24). KC have an essential role in liver fibrosis in mouse models of ASH and NASH, propagating hepatic inflammation via tumor necrosis factor (TNF) and leukocyte recruitment via intercellular adhesion molecule-1 (ICAM-1) and vascular cell adhesion molecule-1 (VCAM-1) (25). On the contrary, predominance of M2-polarized, interleukin (IL)-10-expressing KC, promoting M1 macrophage apoptosis and hepatocyte senescence, is protective in both experimental ALD or NAFLD models (26). An M1-prone profile has been associated not only with liver injury in NASH patients but also with metabolic impairment (insulin resistance and visceral fat deposition) and with portal hypertension in NASH patients (27). Expanded CD11c^+^ CD206^+^ and C-C chemokine receptor-2 (CCR2^+^) macrophage populations in visceral adipose tissue and a higher production of pro-inflammatory cytokines have been observed in NASH patients (28). Moreover, transcription of pro-inflammatory pathways in adipose tissue corresponds to progressive histologic impairment in NASH patients. Central molecules identified in these pathways are IL-8, C-C chemokine ligand-2 (CCL-2), JUN-B, and IL-6, all of which are involved in inflammation (28). CCR2^+^ monocyte-derived macrophages are recruited to the liver (but also to the adipose tissue or atherosclerotic plaques) in metabolic disorders (29), making this pathway an attractive target for inflammatory therapies in NASH.

Monocytes and macrophages have indeed become interesting potential targets for the treatment of NAFLD and ALD. The different mechanisms by which these cells can be targeted include modulation of KC activation, monocyte recruitment in the liver and macrophage polarization and differentiation (30). Evidence from preclinical studies and clinical trials (some of them already in phase II and III) have shown encouraging results in ameliorating steatohepatitis, fibrosis, and the metabolic profile, individuating promising therapeutic candidates.

Granulocytes are also implicated in the onset of fatty liver and steatohepatitis (14). Neutrophils are involved in adipose tissue inflammation, in the induction of insulin resistance and in the progression to steatohepatitis (31–34). There is some evidence that eosinophils, basophils and mast cells may be associated to metabolic impairment, while mast-cell infiltration may also promote liver fibrosis (35–38). Dendritic cells are professional antigen presenting cells that are implicated in the induction of central and peripheral immunological tolerance, in the regulation of the T-cell immune responses, and act as sentinel cells of innate immunity in the recognition of microbial pathogens. These cells are associated to hepatic fibro-inflammatory injury (39, 40).

Considering all these mechanisms, myeloid derived-cells are candidate novel targets for the treatment of NAFLD (41) and ALD (Table 3).

**Table 3:** Summary of the treatments in development for NAFLD (#) and ALD (§).

**Compound**	**Classification**	**Effect**	**Mechanisms of action**
Cenicriviroc #	CCR2-CCR5 dual antagonist	Fibrosis regression Improvement of grade-2 ballooning (42)	Reduction high-sensitivity C-reactive protein, IL-6, IL-1ß, and fibrinogen Reduction of monocyte activation through CCR2-CCR5 blockade (42)
Selonsertib #,§	ASK-1 inhibitor	Fibrosis regression No effect on steatohepatitis No effect on metabolic parameters (43)	Reduction p38 and JNK phosphorylation (44) Inflammatory signaling pathways blockade Macrophage activation impairment (43)
Elafibranor #	Dual PPARα-δ agonist	Resolution of NASH without worsening of fibrosis Regression of fibrosis (if response to treatment) Improvement of serum lipids Improvement of glycaemic control Reduction of calculated overall cardiovascular risk (45)	PPARα: *Control of fatty acid transport and β-oxidation* Dampening of inflammatory response (47, 48) PPARδ: *M2 polarization of KC* (49)
Lanifibranor	Pan-PPAR agonist	*Improvement of liver histology* *Anti-fibrogenic effect* *Improvement of insulin sensitivity and serum triglycerides* *Improvement of body weight and adiposity index* (46)	*Decreased expression of inflammasome components* (50) PPARγ: *Promotion of adipocyte differentiation* (51) Increase of glucose uptake and reduction of triglycerides (52, 53) *Increase of anti-inflammatory cytokines* (54)
Obeticolic acid #,§	Bile acid FXR agonist	Improvement of fibrosis Improvement of steatohepatitis (55, 56) Decrease of HDL No improvement of glycaemic control (55)	*Targets KC* *Decrease of TNF-α and LPS* *Decrease of MCP-1 and IL-10* (57)
BAR501 #	GPBAR-1 agonist	*Reduction of steatosis* *Reduction of inflammation* *Improvement of fibrosis* (58, 59)	*Release of GLP-1* *Modulation of macrophage phenotype* (58, 59)
BI 1467335 #	VAP-1 inhibitor	*Reduction of liver injury* (60)	*Reduction of leucocyte infiltration in the liver during fibrogenesis* (60)
Tipelukast #	Leukotriene receptor antagonist	*Anti-inflammatory and anti-fibrotic properties* *Decrease of serum triglycerides* (61)	*Down-regulation of inflammation-related genes (including CCR2 and MCP-1)* (61)
JKB-121 #	TLR-4 receptor antagonist	*Prevention of LPS-induced inflammatory liver injury in MCDD model* No benefit on human liver disease (62)	*Stimulation of KC activation* (62)
Emricasan #,§	Pan-caspase inhibitor	*Effective in preclinical models of liver injury (including NAFLD and ALD)* (63) Decrease of transaminases in viral hepatitis (64)	*Interference with the signaling cascade of the NLRP-3 inflammasom*e (63)
GR-MD-02 #	Galectin-3 inhibitor	Reduction of portal pressure Reduction of occurrence of esophageal varices (65)	*Interference with fibrogenesis* (66)
HepaStem #	Liver-derived mesenchymal stem cells	Reduction in NAS and fibrosis in mouse model of NASH (67, 68)	*Inhibition DC differentiation* *Inhibition of TNF-α production* *Promotion hepatocyte regeneration* (67)
G-CSF §	G-CSF	Mobilization of hematopoietic stem cells Improvement of liver function and survival in AH	Stimulation of neutrophil function Mobilization of hematopoietic stem cells Induction of liver regeneration (69–71)
DUR-928 §	Small molecule epigenetic regulator	*Anti-fibrotic and anti-inflammatory properties* (72)	*Reduction of MCP-1 and TNF-α* (72)

*NAFLD, Non-alcoholic fatty liver disease; NASH, non-alcoholic steatohepatitis; ALD, Alcoholic liver disease; AH, alcoholic hepatitis; CCR, C-C chemokine receptor; ASK, apoptosis signal-regulating kinase; PPAR, peroxisome proliferator-activated receptor; FXR, farnesoid X receptor; GPBAR, G protein-coupled bile acid receptor; VAP, Vascular adhesion protein-1; TLR, Toll-like receptor; G-CSF, Granulocyte-colony stimulating factor; IL, interleukin; KC, Kupffer cells; TNF, tumor necrosis factor; LPS, lipopolysaccharide; MCP, monocyte chemoattractant protein; GLP, glucagon-like peptide; NLRP, nucleotide oligomerization domain-like receptor family pyrin domain containing protein; DC, dendritic cells. Preclinical data are indicated in cursive; HDL, high-density lipoprotein; MCDD, methionine/choline- deficient diet*.

## Drugs in Development for NASH Treatment

### Cenicriviroc

Cenicriviroc is a CCR2-CCR5 dual antagonist. CCR2 and CCR5 play an important role in macrophage recruitment and polarization (42, 73). CCR2-CCR5 blockade showed anti-inflammatory and anti-fibrotic effects in preclinical models (73–75) and clinical studies (42, 76, 77). The year 1 analysis of a large 2-years phase-2 trial (42) showed a significant decrease in systemic inflammation but could not show a significant improvement in the activity of steatohepatitis and its components (except for ballooning) as assessed by histology. Although the primary endpoint of hepatic histological improvement in NASH Activity Score (NAS) (2) (more than 2 points and no worsening of fibrosis stage) was hence not met, the study did show a significant benefit of cenicriviroc over placebo in terms of regression of fibrosis and amelioration of grade-2 ballooning at histology. As mentioned, the drug was also effective in attenuating the inflammatory signaling. Cenicriviroc was able to induce the reduction of circulating markers of systemic inflammation (such as high-sensitivity C-reactive protein, IL-6, IL-1ß, and fibrinogen) and soluble cluster of differentiation-14 (a marker of monocyte activation) and induced an increase in CCL-2 and CCL-4, confirming potent CCR2-CCR5 blockade. These findings are consistent with previous studies including those conducted in HIV patients (74, 77, 78). Currently, the drug is further being investigated as anti-fibrotic agent in a phase-3 trial with reduction of fibrosis as the primary endpoint.

### Selonsertib

Selonsertib is an apoptosis signal-regulating kinase-1 (ASK-1) inhibitor. ASK-1 is a ubiquitously expressed serine/threonine kinase, which is activated by oxidative stress to promote hepatocellular apoptosis, inflammation and fibrosis, via downstream phosphorylation of p38 and Jun N-terminal kinases (JNK). Both p38 and JNK have well-characterized roles, not only in hepatocytes but also in other cell types, including macrophages (79–81). KC are indeed activated, among others, by p38 and JNK and blocking the inflammatory signaling pathways of KC was shown to reduce inflammation and fibrogenesis in NASH (81). Therefore, it is plausible that Selonsertib also interferes with macrophage activation (43). Selonsertib was tested in a small 6-months trial in combination with or without Simtuzumab in an anti-fibrotic strategy. Selonsertib was superior to placebo (Simtuzumab was considered a placebo given that other Simtuzumab trials appeared negative) in terms of fibrosis regression, without an effect on steatohepatitis or on the metabolic features. Selonsertib is tested in 2 Phase-3 trials, one in F4 and one in F3 patients (43). The trial in F4 patients was recently reported to be negative on the pre-specified week 48 primary endpoint of a ≥ 1-stage histologic improvement in fibrosis without worsening of NASH. Selonsertib was generally well-tolerated and safety results were consistent with prior studies. The trial was discontinued. The trial in patients with F3 is still ongoing (82).

### Peroxisome Proliferator-Activated Receptors (PPAR) Agonists

PPARs are ligand-activated nuclear receptors that function as master regulators in adipose tissue and the liver, controlling insulin sensitivity, glucose and lipid metabolism, inflammation and fibrogenesis (83, 84). There are three isoforms of PPARs. The PPARα isoform is highly expressed in hepatocytes and controls fatty acid transport and β-oxidation and dampens the inflammatory response (47). The PPARγ isoform is highly expressed in adipose tissue; its activation promotes adipocyte differentiation, increases glucose uptake and triglyceride storage (hence reducing free fatty acid flux to the liver), and increases secretion of the anti-inflammatory cytokines like adiponectin. The PPARδ isoform contributes to the regulation of glucose and lipid metabolism. Of note, PPARδ exerts an anti-inflammatory effect in the liver by skewing M2 polarization of KC and decreases the expression of inflammasome components [nucleotide oligomerization domain-like receptor family, pyrin domain containing-3 (NLRP-3), caspase-1, and IL-1] under stimulus of saturated fatty acids and lipopolysaccharides. PPARs also interact with hepatic stellate cells (HSC) to regulate fibrosis: PPARγ and PPARδ are expressed at various levels in HSC, which contribute to liver fibrosis, while PPARγ holds HSC in a quiescent non-fibrogenic state (46).

PPARα agonists like fibrates failed to show a histological benefit in NASH (85). However, recent data showed that PPARα expression is inversely correlated to the severity of NASH and that NASH improvement is associated with increased PPARα expression, giving rationale to a PPARα-targeted treatment despite the negative data with fibrates (86). Several multi- or pan-agonists are in development and, by means of the δ isoform, are likely to act on macrophages. Elafibranor, a dual PPARα-δ agonist, was able to induce resolution of NASH without worsening of fibrosis in significantly more patients compared to placebo if baseline NASH was sufficiently severe. Moreover, it was shown to reduce fibrosis in those patients that responded to treatment (45). Additionally, it improved serum lipids and glycaemic control, reducing the calculated overall cardiovascular risk (45). Elafibranor is now in phase-3 and the first part of the cohort needed for the interim analysis has recently been fully recruited.

Lanifibranor is a next-generation pan-PPAR agonist. In different preclinical models of NASH, Lanifibranor induced an improvement of liver histology (including an anti-fibrogenic effect) and of the metabolic profile (ameliorated insulin sensitivity, body weight, adiposity index and serum triglycerides). Moreover, Lanifibranor inhibited the expression of pro-fibrotic and inflammasome-related genes while increasing the expression of β-oxidation-related and fatty acid desaturation-related genes in both the methionine/choline-deficient diet (MCDD) and in the foz/foz model. Additionally, in the foz/foz model it showed a reduced macrophage recruitment (46). Lanifibranor is currently being evaluated in a phase-2 study.

### Farnesoid X Receptor (FXR) Agonist

FXR plays an important role in bile acid metabolism, but also impacts on several metabolic, and fibrogenic pathways (55). Obeticholic acid (OCA) is a potent bile acid FXR agonist already licensed for the treatment of primary biliary cholangitis and under investigation in the setting of NASH. Preclinical studies have shown that OCA also targets KC, as shown by the dose-dependent inhibition of TNF-α and bacterial lipopolysaccharide (LPS)-stimulated expression of monocyte chemoattractant protein-1 (MCP-1) in KC (57). Moreover, this effect of OCA on KC translates in a decrease of not only pro-inflammatory cytokines, but also of anti-inflammatory cytokines, such as IL-10. In a phase-2 study OCA showed a significant response—defined as a NAS reduction of ≥2 points–compared to placebo, as well as a beneficial effect on fibrosis (which was a secondary study endpoint). These results were, however, associated with a decrease in high-density lipoprotein (HDL) levels and a lack of improvement of glycaemic control (87). At this moment, the study has proceeded to a phase 3 study. The recently released interim analysis showed that in the primary efficacy analysis (Intent-to-Treat), once-daily OCA 25 mg met the primary endpoint of fibrosis improvement (≥1 stage) with no worsening of NASH. Moreover, a greater proportion of patients treated with OCA compared to placebo achieved the primary endpoint of NASH resolution with no worsening of liver fibrosis, although statistical significance was not reached (56).

### G Protein-Coupled Bile Acid Receptor-1 (GPBAR-1) Agonist

GPBAR-1 is a G-protein coupled receptor, activated by secondary bile acids. GPBAR-1 is expressed in various cells types in the intestine, the adipose tissues and non-parenchymal liver cells, particularly KC. The activation of this receptor in the intestine causes the release of glucagon-like peptide-1 (GLP-1). Moreover, this receptor is highly expressed by monocytes and macrophages and its activation counter-regulates the innate immune response in the intestine and liver. Activation of GPBAR-1 is also able to modulate the macrophage phenotype from a classically activated (M1) to an alternatively activated (M2) phenotype. BAR501 is a non-bile acid, selective GPBAR-1 ligand that has been shown effective in reducing steatosis, inflammation and fibrosis in preclinical models of NASH (58, 59) and is currently under development for the treatment of NASH.

### Vascular Adhesion Protein-1 (VAP-1) Inhibitors

The semicarbazide-sensitive amine oxidase (SSAO)/VAP-1 is a homodimeric glycoprotein adhesion molecule that is widely expressed in the vascular system. During inflammation this complex facilitates leukocyte recruitment through its SSAO component and its activation promotes liver inflammation and fibrosis. Moreover, its soluble variant showed a correlation with NAFLD severity in humans. BI1467335 is an oral small molecule SSAO/VAP-1 inhibitor that was shown effective in reducing liver injury in rodents. VAP-1 inhibition blunted leucocyte (including macrophages and other myeloid cells) infiltration in the liver during fibrogenesis (60). A phase-2 clinical trial in patients with NASH was started in 2017 (88).

### Tipelukast

Tipelukast, also known as MN-001, is an orally bioavailable small molecule leukotriene receptor antagonist. The molecule was shown to be anti-fibrotic and anti-inflammatory in preclinical models and exerts these effects through several mechanisms, including: leukotriene (LT) receptor antagonism, inhibition of phosphodiesterases (PDE) (mainly 3 and 4), and inhibition of 5-lipoxygenase (5-LO). It has also been shown to down-regulate expression of genes that promote inflammation, including CCR2 and MCP-1. A phase-2 open-label study to evaluate the effectiveness, safety, tolerability and pharmacokinetics of tipelukast, including its effects on HDL function and serum triglyceride levels in patients with NASH/NAFLD and hypertriglyceridemia, is ongoing (89). The interim analysis showed a significant decrease of serum triglycerides, which was a primary endpoint (61).

### Toll-Like Receptor-4 (TLR-4) Receptor Antagonist

JKB-121 is a long-acting small molecule that functions as a TLR-4 receptor antagonist. TLRs are expressed by KC and are able to stimulate their activation upon infectious and non-infectious threats in order to induce a immunogenic T-cell response (90). It has been shown that JKB-121 prevents LPS-induced inflammatory liver injury in a MCDD rat model of NAFLD. Although the preclinical data were promising and were based on a solid rationale, the results of a phase-2 study failed to show a beneficial effect on liver disease (62).

### Caspase Inhibitors

Inhibition of caspases attenuates inflammatory and apoptotic processes by interfering with the signaling cascade of the NLRP-3 inflammasome, which was shown to be activated in KC in preclinical models of NASH and ALD (63). Emricasan, a pan-caspase inhibitor, was shown to be effective in lowering transaminase levels and attenuating fibrosis in a preclinical animal model (91). Interestingly, this molecule was already shown to decrease transaminase levels in chronic hepatitis C patients (64). The compound is currently in phase 2 for the treatment of NASH.

### Galectin-3 Inhibitor

Galectin-3 is a protein expressed predominantly in immune cells that recognizes and binds to galactose residues and is an essential protein in liver fibrogenesis (66). GR-MD-02 is a galectin-3 inhibitor that is currently undergoing a phase-2b trial in NASH patients with fibrosis/cirrhosis. The interim analysis of this study (65) suggests a clinical improvement in cirrhotic patients: significant decrease in portal pressure and a reduction in the development of newly formed esophageal varices.

### Cell-Based Therapy

Another frontier in NASH treatment is cell-based therapy, which is currently given full consideration for application in clinical trials. HepaStem are liver-derived mesenchymal stem cells (MSC) with regenerative, anti-fibrotic, and anti-inflammatory potential. MSC can affect monocyte and DC recruitment, differentiation, maturation and function (92, 93). HepaStem have been shown to inhibit T-cell proliferation and activation as well as DC differentiation, maturation and production of TNF-α *in vitro* and can promote hepatocyte regeneration by inhibiting HSC (67). In a mouse model of NASH HepaStem were shown to induce a reduction in NAS and fibrosis (67, 68). In humans, a phase-2 trial is ongoing in patients with acute liver failure (94).

## Drugs in Development for ALD Treatment

Macrophages are potential targets for the treatment of ALD (18). It is a well-established concept that alcohol abstinence is the cornerstone in the treatment of ALD. Alcohol abstinence *per se* can influence macrophage function in terms of cytokine production (95) and phenotype switching (96). Anti-inflammatory treatments targeting macrophage function, such as treatment with corticosteroids and pentoxyfyllin (a phosphodiesterase inhibitor) have long been evaluated for ALD (18). Corticosteroids constitute the standard treatment of severe AH and pentoxyfillin can be used for this indication in those patients with contraindications to corticosteroids (3, 20). In contrast, anti-TNFα antibodies did not show effectiveness in the treatment of AH and yielded a higher probability of severe infections and a higher mortality (97). Macrophages, however, remain a candidate target for the treatment of ALD, particularly AH, its most severe form. Combining biologicals, small-molecule drugs and anti-oxidant therapies targeting macrophage function and phenotype could provide an added therapeutic benefit (5). Therefore, new drugs targeting macrophages are currently being evaluated in clinical trials (Table 3).

### Selonsertib

Besides the ongoing studies in NAFLD patients, mentioned above, the ASK-1 inhibitor Selonsertib is also currently under investigation in the setting of AH. As previously reported, the downstream effect of ASK-1 inhibition would likely also affect macrophage activation (43). A phase-2 study comparing the effect of Selonsertib with prednisolone compared to prednisolone alone in AH has recently completed recruitment.

### FXR Agonists

The FXR agonist OCA is another molecule in development for both NAFLD and ALD. A phase-2 double-blind, placebo-controlled trial of OCA in patients with moderate to severe AH is currently ongoing to evaluate a possible reduction in Model For End-Stage Liver Disease (MELD) score as a measure of effectiveness, as well as the incidence of serious adverse events during treatment.

### Granulocyte Colony-Stimulating Factor (G-CSF)

This cytokine is a potent stimulus of neutrophil function and is able to mobilize hematopoietic stem cells and induce liver regeneration. G-CSF was safe and effective in the mobilization of hematopoietic stem cells and improved liver function and survival in patients with severe alcoholic hepatitis in small trials (69–71). These encouraging results need to be confirmed in larger studies (98).

### Caspase Inhibitors

As mentioned above, inhibition of caspases attenuates inflammatory and apoptotic processes by interfering with the signaling cascade of the NLRP-3 inflammasome, which was shown to be activated in KC in both mouse models of ALD and a human cohort (99–101). Moreover, alcohol exposure was shown to cause hepatocytes to release extracellular vesicles in a caspase-dependent manner to elicit apoptosis and macrophage activation (102). Based on the positive data in NAFLD, Emricasan, a pan-caspase inhibitor, has also been evaluated in the setting of ALD. A phase-2 clinical trial concluded that Child Pugh A and B cirrhotic patients with a baseline MELD ≥15 showed significantly improved liver function compared to placebo (103).

### Small Molecule Epigenetic Regulators

DUR-928 is an endogenous, orally bio-available small molecule that modulates the activity of various nuclear receptors that play an important regulatory role in lipid homeostasis, inflammation and cell survival. It has been demonstrated in mice models of NASH that this molecule exerts anti-fibrotic and anti-inflammatory effects and is able to reduce hepatic transcripts of TNF-α and MCP-1 in a dose-dependent manner (72). DUR-928 is currently being investigated in a phase-2, open-label, dose-escalation study in AH.

## Conclusions

Fatty liver and steatohepatitis (alcoholic and non-alcoholic) constitute a spectrum of highly prevalent liver conditions with a possibly unfavorable outcome, for which the treatment is an unmet medical need. A plethora of clinical trials, many of which acting on inflammatory processes, has been set up in an attempt to resolve this issue. Myeloid-derived cells are promising candidate targets in the pharmacological treatment of these diseases. The results of the phase-3 trials are expected by 2020 and will likely change the scene in the treatment of these diseases.

## Author Contributions

LV and SF conceived the paper. LV and SF wrote the paper with contribution of MVH and JW. LV and MVH designed the figures. SF supervised the paper. All authors contributed to manuscript revision, read, and approved the submitted version.

### Conflict of Interest Statement

SF has a senior clinical research mandate from the Fund for Scientific Research (FWO) Flanders (1802154N) and has acted as an advisor and/or lecturer for Roche, Gilead, Abbvie, Bayer, BMS, MSD, Janssen, Actelion, Astellas, Genfit, Inventiva, and Intercept. LV has acted as an advisor for Inventiva, Abbvie, and Bayer. SF was a partner in the European Commission projects Hepadip (contract LSHM-CT-2005-018734) and Resolve (Contract FP7-305707) and is a partner in the Innovative Medicines Initiative 2 Joint Undertaking LITMUS consortium. The remaining authors declare that the research was conducted in the absence of any commercial or financial relationships that could be construed as a potential conflict of interest.
